# Supplementary material for: Synthesis, Characterization, Cytotoxicity, Cellular Imaging, Molecular Docking, and ADMET Studies of Piperazine-Linked 1,8-Naphthalimide-Arylsulfonyl Derivatives
Source: Int J Mol Sci. 2024 Jan 15;25(2):1069. doi: 10.3390/ijms25021069 (PMC10816875; doi:10.3390/ijms25021069)
Supplement: Supplementary file 1 [file ijms-25-01069-s001.zip › ijms-2782220-supplementary.pdf]

Synthesis, Characterization, Cytotoxicity, Cellular Imaging, Molecular Docking, and ADMET Studies of Piperazine-Linked 1,8-Naphthalimide-Arylsulfonyl Derivatives

Ashanul Haque [1,2], Khalaf M. Alenezi [1,2], Ahmed Al-Otaibi [1,2], Abdulmohsen Khalaf Dhahi Alsukaibi [1,2], Ataur Rahman [3], Ming-Fa Hsieh [4,*], Mei-Wen Tseng [4] and Wai-Yeung Wong [5,*]

1 Department of Chemistry, College of Science, University of Ha'il, Ha'il 81451, Saudi Arabia
2 Medical and Diagnostic Research Centre, University of Ha'il, Ha'il 55473, Saudi Arabia
3 Jamia Senior Secondary School, Jamia Millia Islamia, New Delhi 110025, India
4 Department of Biomedical Engineering, Chung Yuan Christian University, 200 Chung Pei Road, Chung Li District, Taoyuan City 32023, Taiwan
5 Department of Applied Biology and Chemical Technology, The Hong Kong Polytechnic University, Hung Hom, Kowloon, Hong Kong, China
* Correspondence: mfhsieh@cycu.edu.tw (M.-F.H.); wai-yeung.wong@polyu.edu.hk (W.-Y.W.)

Supporting Information

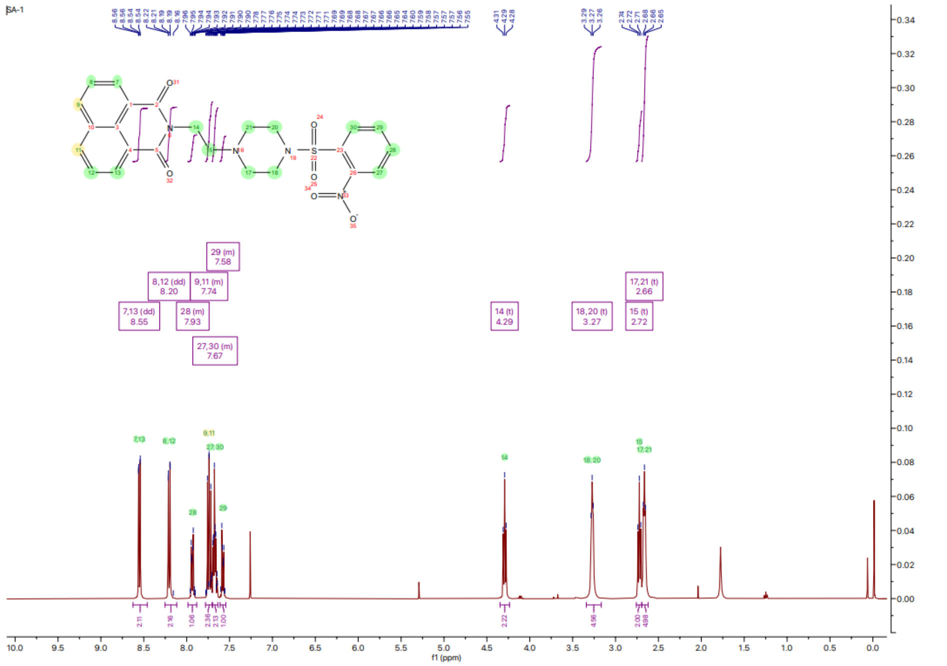

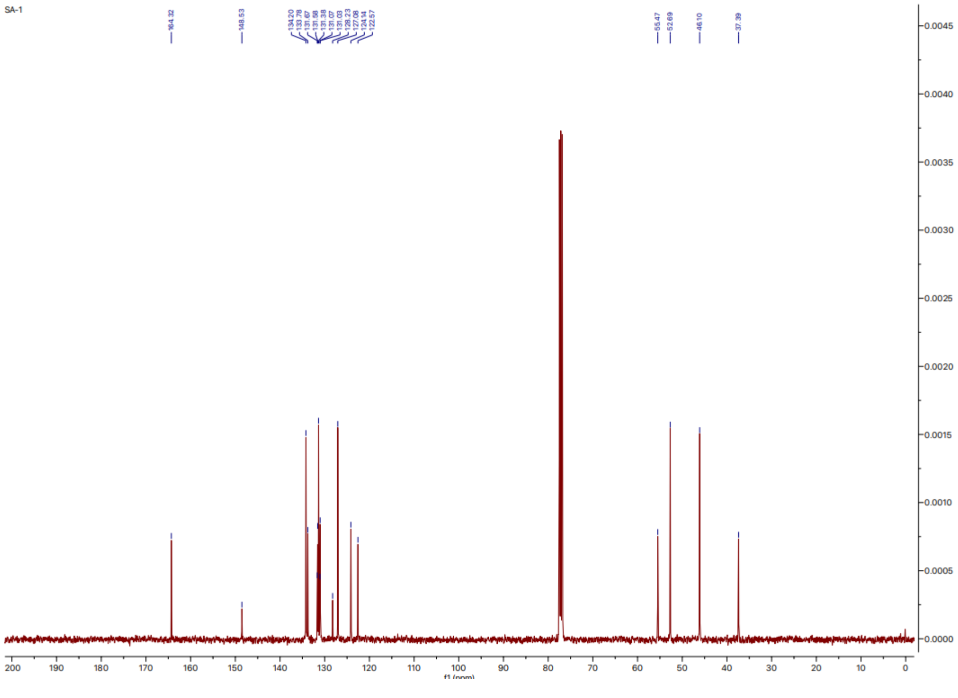

Figure S1: ^{1}H- and ^{13}C-NMR spectra of compound (**SA1**).

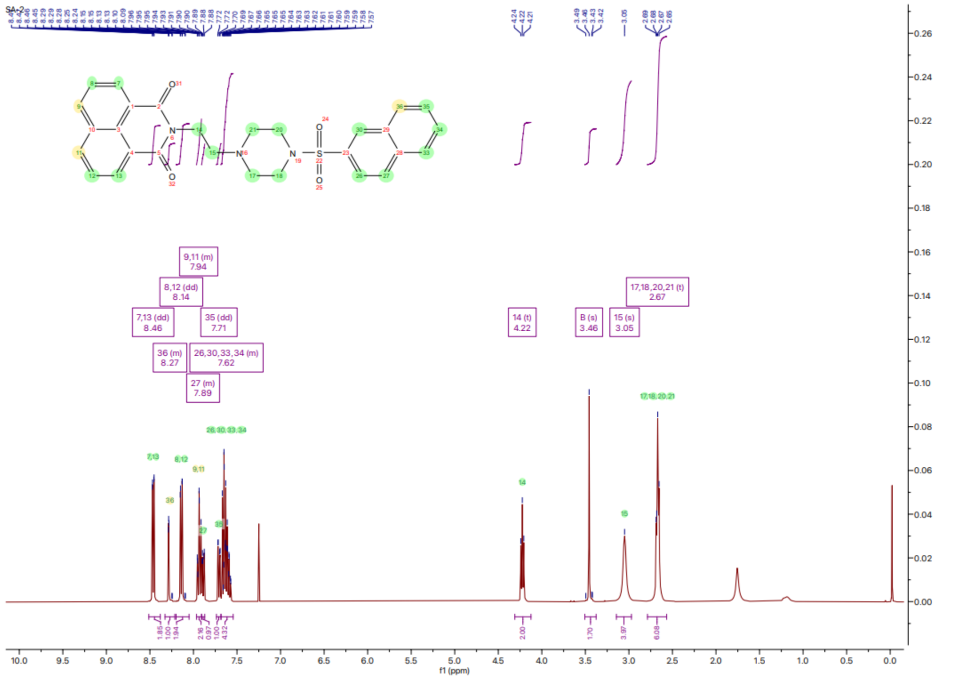

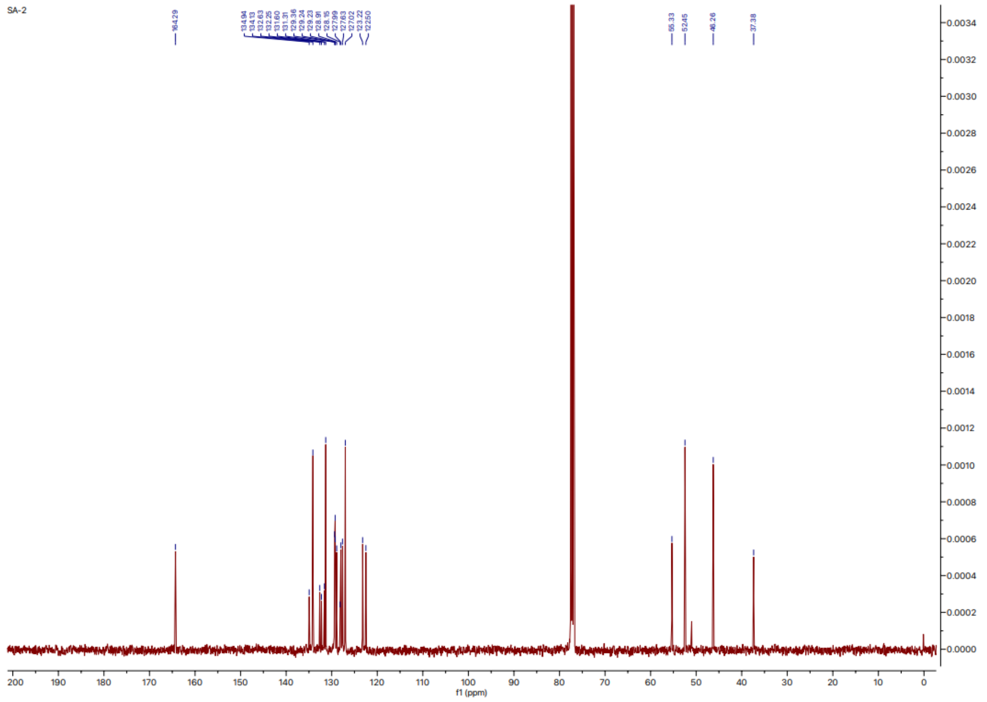

Figure S2: ^{1}H- and ^{13}C-NMR spectra of compound (**SA2**).

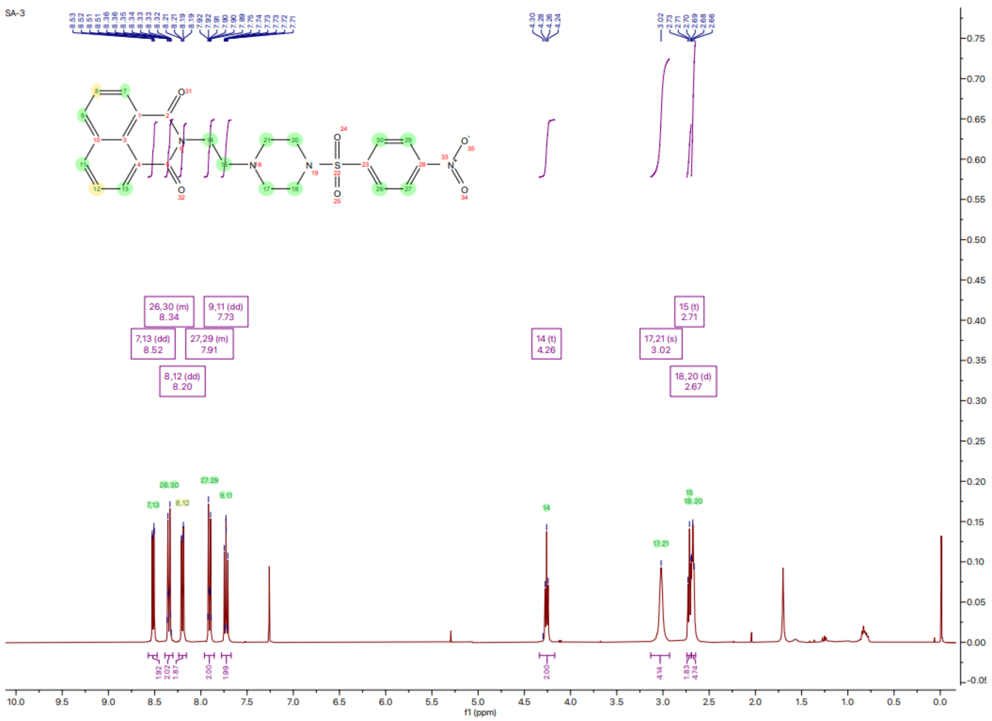

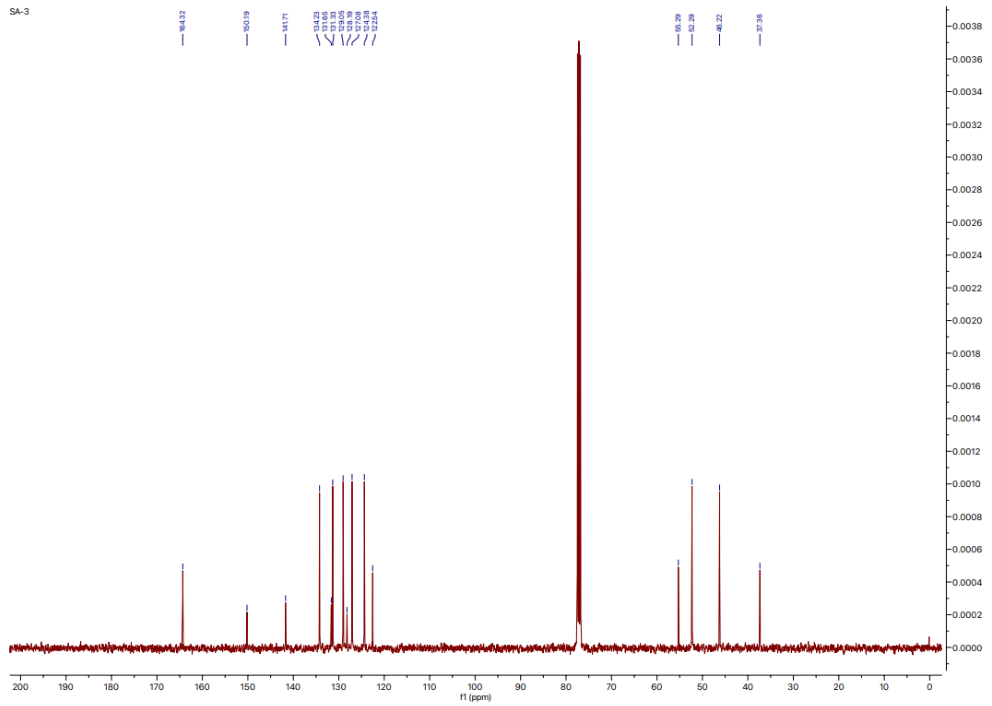

Figure S3: ^{1}H- and ^{13}C-NMR spectra of compound (**SA3**).

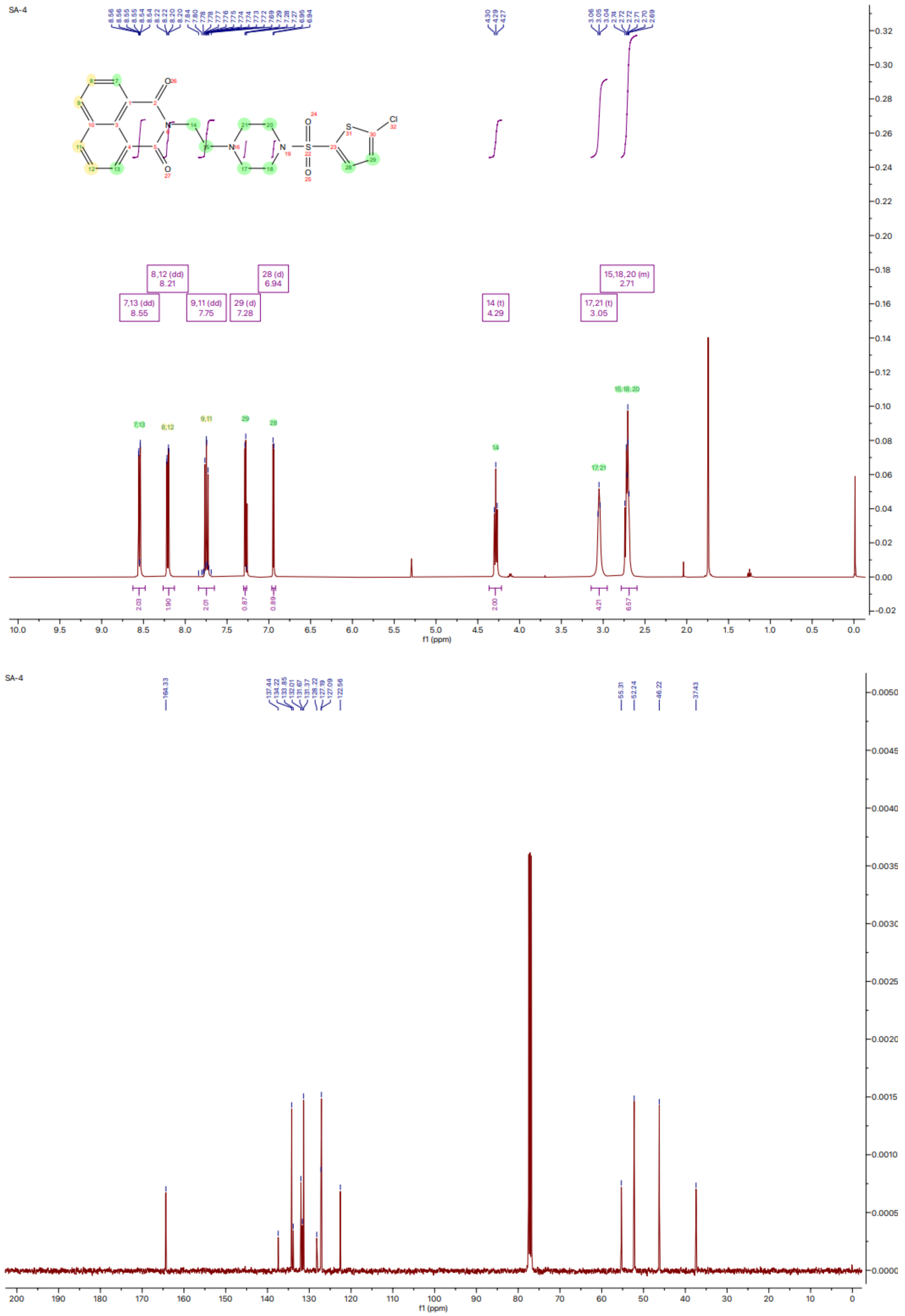

Figure S4: ^{1}H- and ^{13}C-NMR spectra of compound (**SA4**).

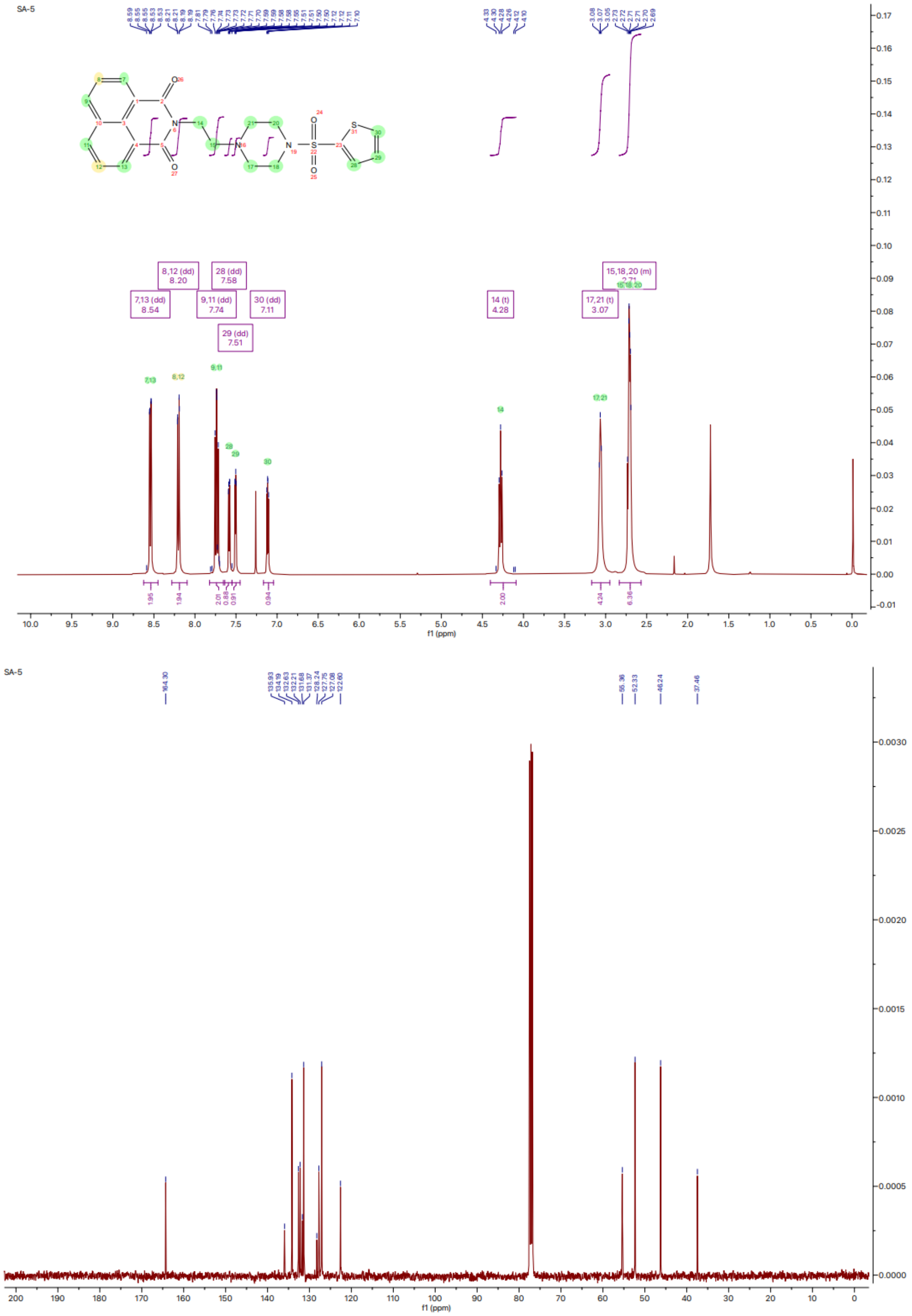

Figure S5: ^{1}H- and ^{13}C-NMR spectra of compound (**SA5**).

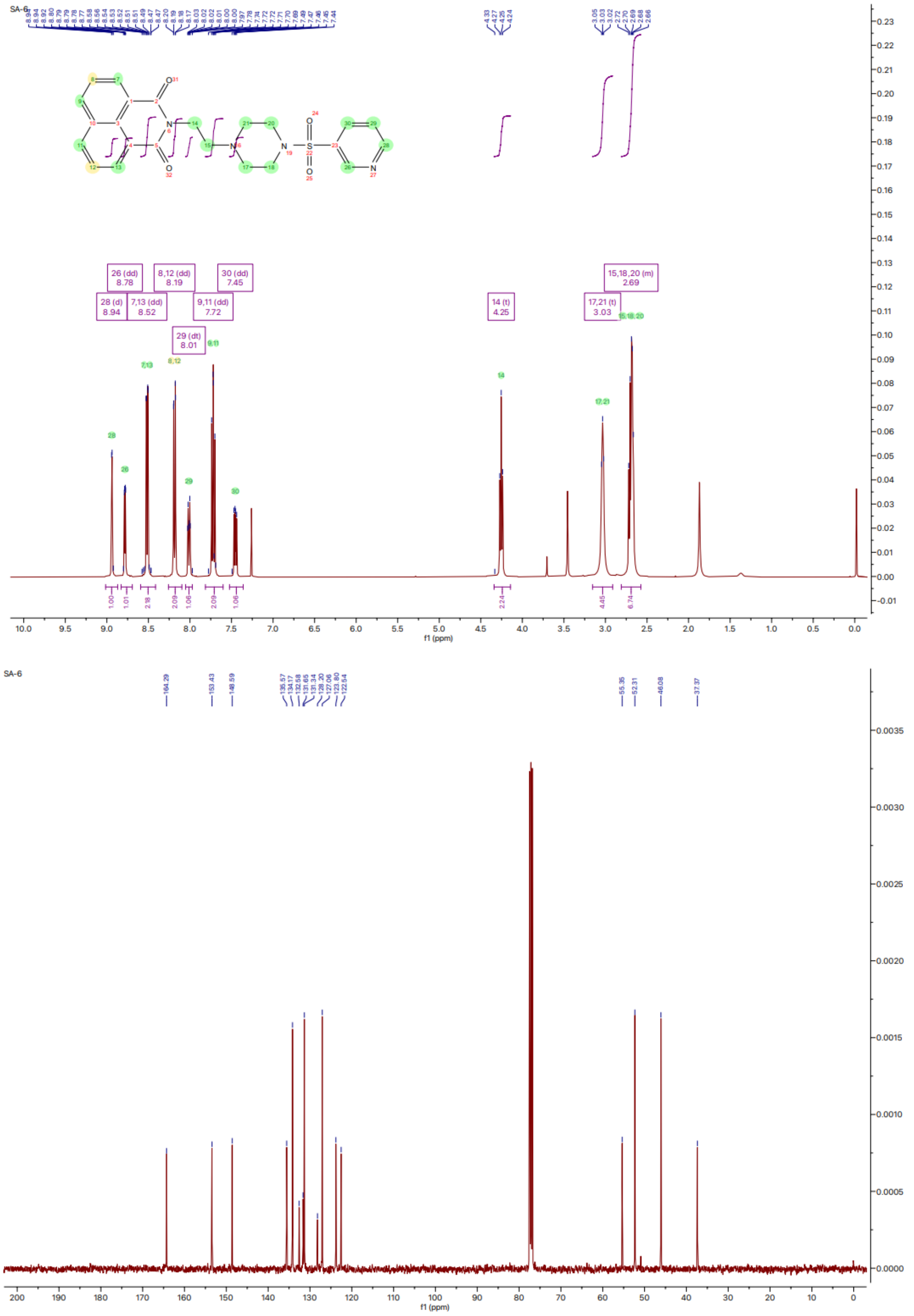

Figure S6: ^{1}H- and ^{13}C-NMR spectra of compound (**SA6**).

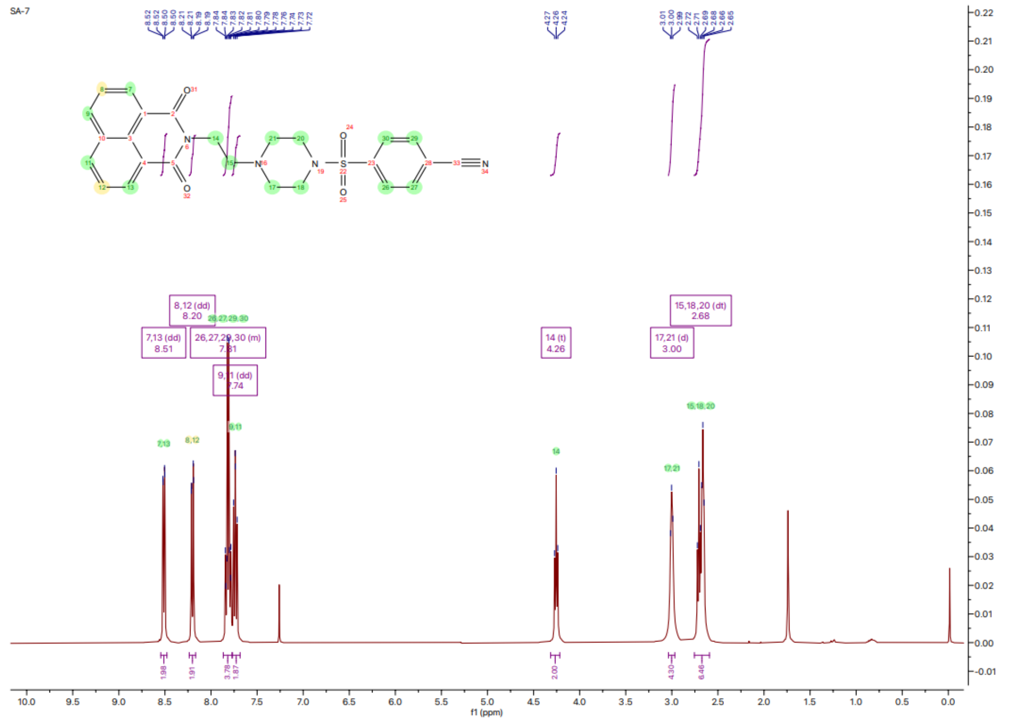

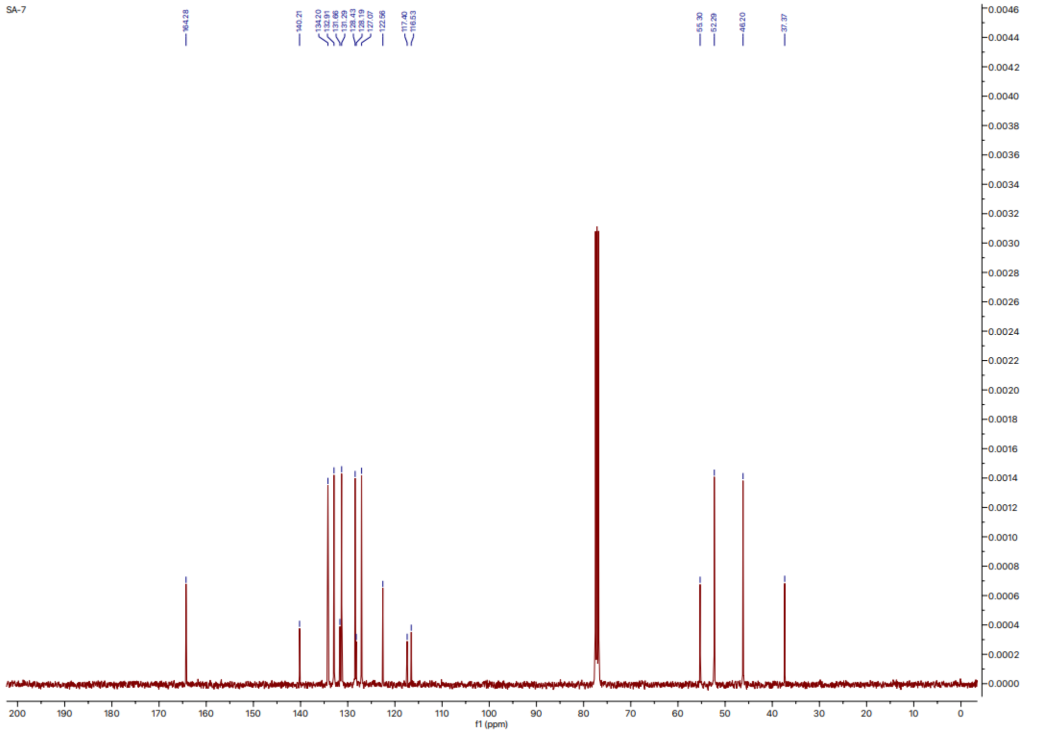

Figure S7: ^{1}H- and ^{13}C-NMR spectra of compound (**SA7**).

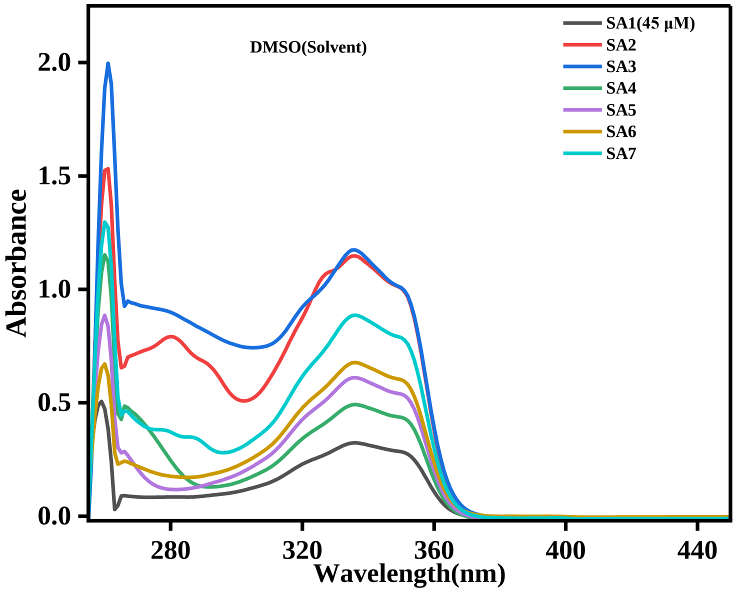

Figure S8: Absorption spectra of **SA1-SA7** in DMSO at RT.

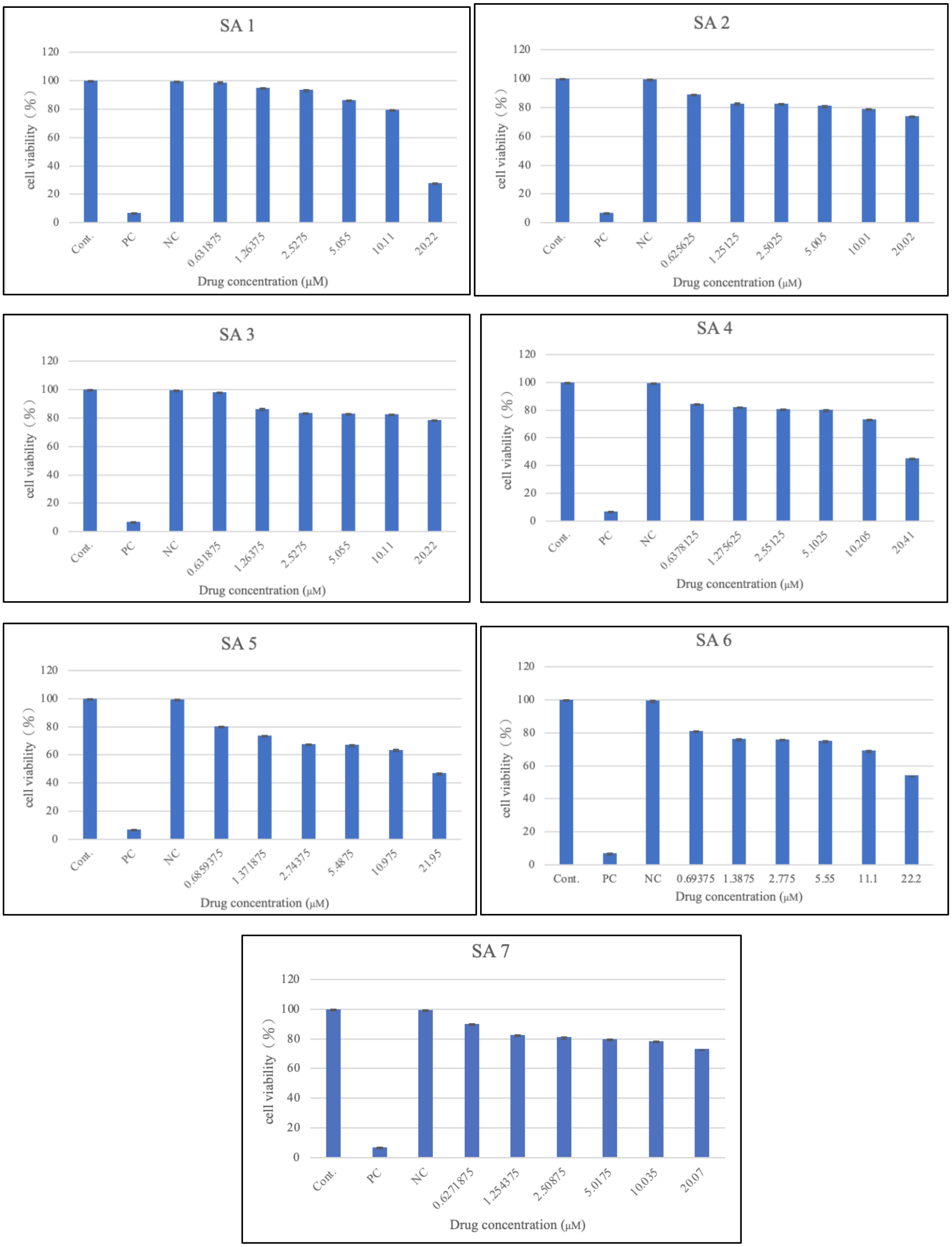

Figure S9: % Cell viability of the **SA1-SA7** against mouse breast cancer 4T1 cell line.

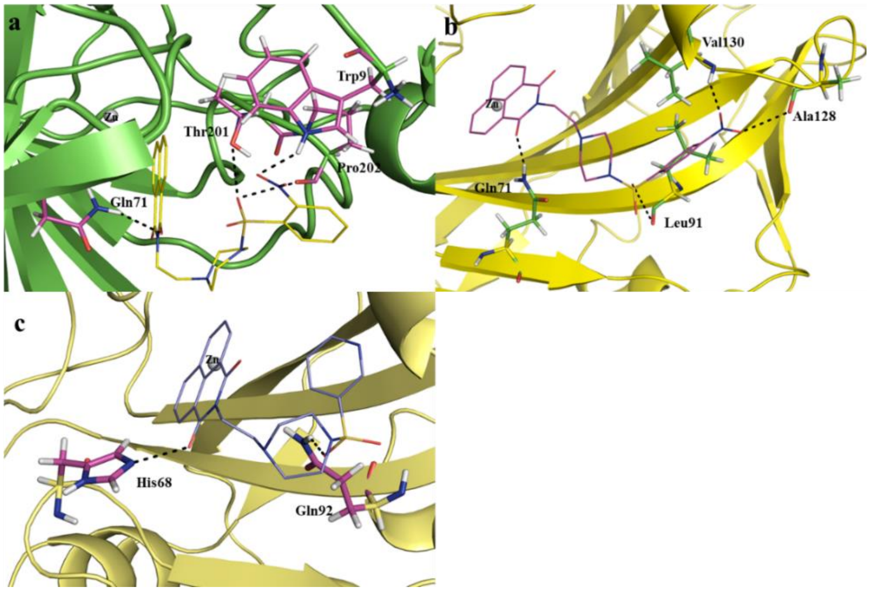

Figure S10: Docking results showing the binding residues of CAIX protein with compounds SA1, SA3, and SA6 (denoted as a-c, respectively). H-bonds are shown in black dotted lines.

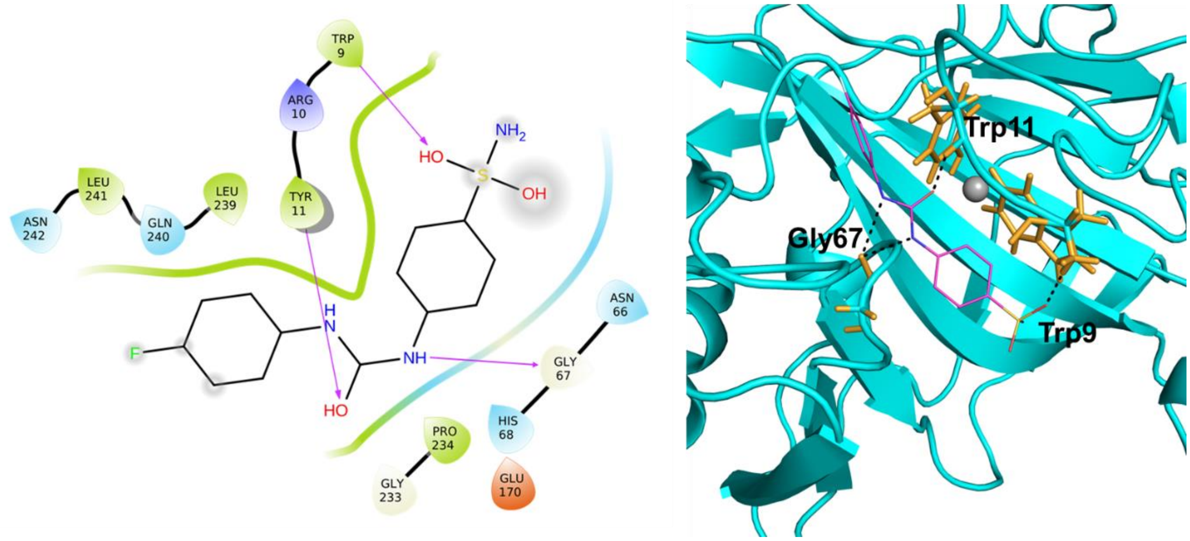

Figure S11: Docking results showing the binding residues of CAIX protein with inhibitor SLC-0111 (4-(3-(4-fluorophenyl)ureido)benzenesulfonamide).

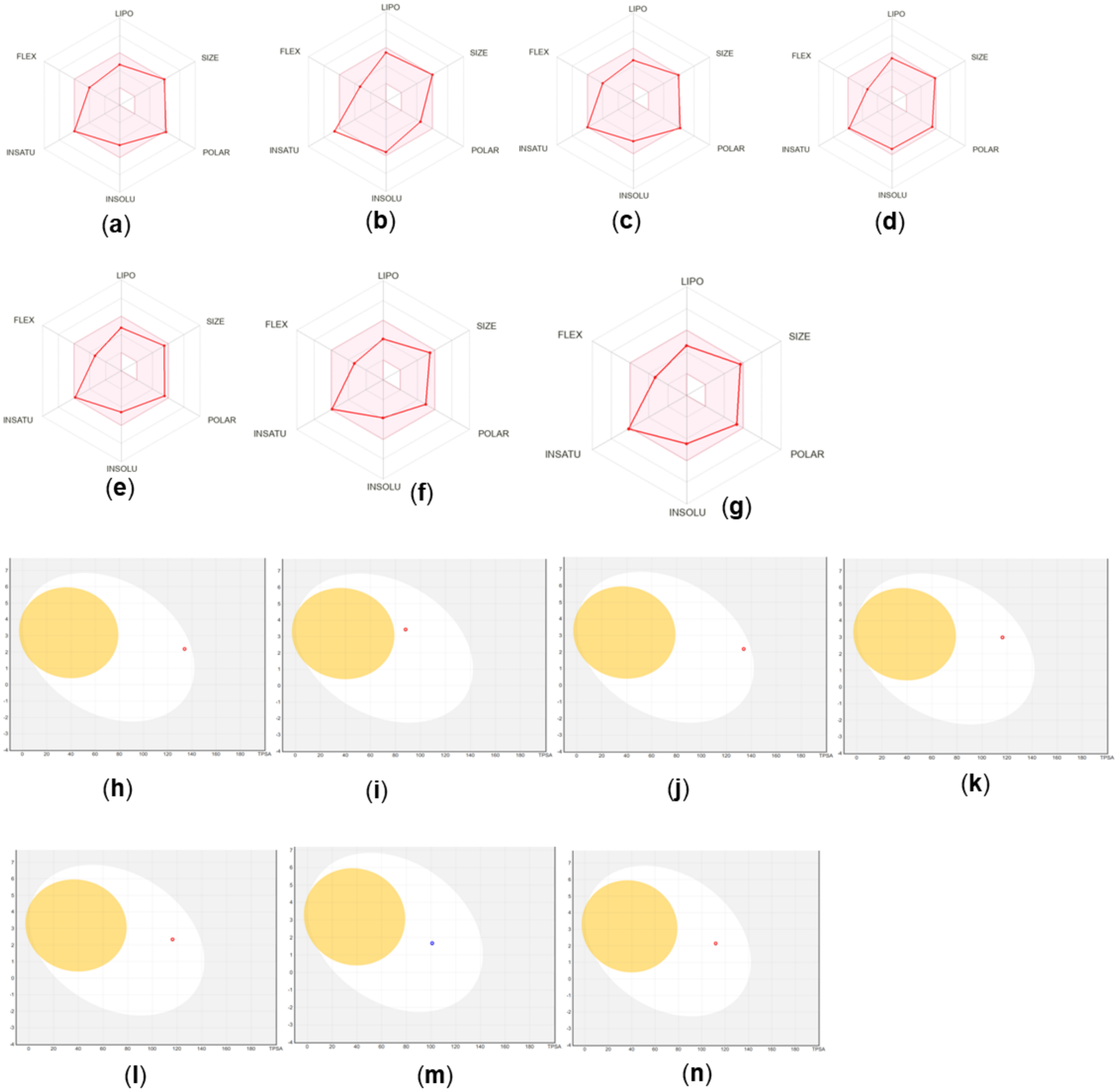

Figure S12: Predicted radar plots (a-g) and BOILED-Egg diagram (h-n) of compounds (**SA1-SA7**). Plots were obtained using the SwissADME web tool. Typical values for a molecule to fall the pink area are as follows: lipophilicity = −0.7 to + 5.0; size: 150 g/mol < molecular weight < 500 g/mol; polarity: 20 Å^2 < topological polar surface area (TPSA) < 130 Å^2; insolubility: 0 < LogS < 6; and INSATU (insaturation): 0.25 < fraction of carbons in sp^3 hybridization < 1; and flexibility: 0 < number of rotatable bonds < 9.

Table S1: ADME (Absorption, Distribution, Metabolism, and Excretion) properties were calculated for all compounds using the QikProp module of Schrödinger.

S. No.	QPlogPC16	QPlogPoct	QPlogPw	QPlogPo/w	QPlogS	CIQPlogS	QPlogHERG	QPPCaco	QPlogBB	QPPMDCK	QPlogKp	QPlogKhsa	%HOA	PSA	RO3
SA1	14.976	23.164	14.353	1.783	-2.36	-4.896	-7.005	42.409	-1.25	18.011	-5.095	-0.454	66.512	132.54	0
SA2	16.172	23.477	14.052	3.416	-4.097	-5.707	-8.007	187.578	-0.595	90.346	-3.525	0.095	87.633	94.159	0
SA3	15.288	22.933	14.518	1.6	-2.791	-4.896	-7.213	18.92	-1.723	7.574	-5.849	-0.412	59.167	139.171	1
SA4	14.64	22.105	12.824	2.781	-3.539	-5.204	-6.954	147.329	-0.394	286.431	-4.355	-0.252	82.035	95.259	0
SA5	14.015	21.461	13.043	2.268	-2.745	-4.472	-6.999	147.221	-0.534	117.474	-4.198	-0.383	79.029	95.351	0
SA6	14.17	22.152	14.832	1.319	-1.876	-3.729	-7.108	85.755	-0.886	38.781	-4.579	-0.746	69.272	107.307	0
SA7	15.218	23.054	15.003	1.512	-3.512	-5.239	-7.365	33.14	-1.466	13.874	-5.288	-0.568	63.009	119.999	0

QPlogPo/w (octanol/water partition coefficient); Plog S (aqueous solubility); QPlog Khsa (Binding to human serum albumin); QPPCaco (Apparent Caco cell permeability); Brain/blood partition coefficient (QPlogBB); Percentage human oral absorption (%HOA); Polar surface area (PSA)
